# Into the Wild: Neuroergonomic Differentiation of Hand-Held and Augmented Reality Wearable Displays during Outdoor Navigation with Functional Near Infrared Spectroscopy

**DOI:** 10.3389/fnhum.2016.00216

**Published:** 2016-05-18

**Authors:** Ryan McKendrick, Raja Parasuraman, Rabia Murtza, Alice Formwalt, Wendy Baccus, Martin Paczynski, Hasan Ayaz

**Affiliations:** ^1^Psychology Department, Human Factors and Applied Cognition, George Mason UniversityFairfax, VA, USA; ^2^School of Biomedical Engineering, Science and Health Systems, Drexel UniversityPhiladelphia, PA, USA; ^3^Department of Family and Community Health, University of PennsylvaniaPhiladelphia, PA, USA; ^4^Division of General Pediatrics, Children’s Hospital of PhiladelphiaPhiladelphia, PA, USA

**Keywords:** fNIRS, situation awareness, mental workload, spatial navigation, working memory, head-mounted display, neuroergonomics

## Abstract

Highly mobile computing devices promise to improve quality of life, productivity, and performance. Increased situation awareness and reduced mental workload are two potential means by which this can be accomplished. However, it is difficult to measure these concepts in the “wild”. We employed ultra-portable battery operated and wireless functional near infrared spectroscopy (fNIRS) to non-invasively measure hemodynamic changes in the brain’s Prefrontal cortex (PFC). Measurements were taken during navigation of a college campus with either a hand-held display, or an Augmented reality wearable display (ARWD). Hemodynamic measures were also paired with secondary tasks of visual perception and auditory working memory to provide behavioral assessment of situation awareness and mental workload. Navigating with an augmented reality wearable display produced the least workload during the auditory working memory task, and a trend for improved situation awareness in our measures of prefrontal hemodynamics. The hemodynamics associated with errors were also different between the two devices. Errors with an augmented reality wearable display were associated with increased prefrontal activity and the opposite was observed for the hand-held display. This suggests that the cognitive mechanisms underlying errors between the two devices differ. These findings show fNIRS is a valuable tool for assessing new technology in ecologically valid settings and that ARWDs offer benefits with regards to mental workload while navigating, and potentially superior situation awareness with improved display design.

## Introduction

The availability and use of highly mobile computing devices is increasing. Examples include fitness trackers, smartwatches, and smartphones; however, there are also devices such as Google Glass, Occulus Rift and Microsoft Hololens which promise not just mobile computing but the coexistence of real world objects with supplementary computer generated objects (i.e., augmented reality; Azuma et al., 2001). Augmented reality wearable displays (ARWD) are already being put into service by the National Aeronautics and Space Administration (NASA). It is believed that these devices will help astronauts on the international space station improve their training and performance in highly demanding situations (Schierholz et al., 2015). While it is clear that having a hands-free display can improve physical ergonomics, especially when both hands are required for adequate task execution, ARWDs could also enhance cognitive ergonomics through augmentation of mental workload and situation awareness.

Ideal task performance is dependent on optimizing mental workload. Mental workload refers to the limited information processing capacity of the brain that is demanded by a task (Parasuraman et al., 2008). When demands exceed the brains maximum information processing capacity, further increases in mental workload lead to ever increasing decrements in performance (Hancock and Parasuraman, 1992). This can be realized as incorrect responses, missed responses or even the “shedding” of secondary tasks (Wickens et al., 2013). ARWDs have the potential to reduce mental workload by reducing the distance and time between visual fixations. Reducing fixation time and distance could reduce the amount of information needed to be held in working memory. For example, during simulated emergency braking, drivers using Google Glass to send text messages experienced less mental workload relative to drivers using a smartphone (Sawyer et al., 2014). ARWDs have also been used to improve operator comfort and procedure efficiency during cardiac surgery (Opolski et al., 2015). An ARWD allowed cardiologists to view reconstructed tomographic images while performing catheterization, improving landmark visualization and verification of surgical tools.

Situation awareness, the perception of critical information (stage 1), comprehension of its meaning (stage 2), and the projection of this information into the future (stage 3; Endsley, 1995a) is also critical for complex task performance (Wickens et al., 2013). High situation awareness, while not guaranteeing successful performance, increases the probability of successful performance. Like mental workload, situation awareness is dependent on working memory and highly dependent on attention (Endsley, 1995a). In this regard ARWDs have the potential to both enhance and degrade situation awareness. ARWDs may enhance situation awareness by freeing up working memory capacity. Conversely, ARWDS may reduce situation awareness from degradation of divided attention. Divided attention relates to the optimal allocation of attention to different inputs by splitting or rapidly shifting the focus of attention (Parasuraman, 1998). The compellingness of ARWD symbology is more likely to exogenously capture the focus of attention and hold it (Thomas and Wickens, 2001, 2004). This results in increased focused attention to display elements, and reduced or eliminated attention to task relevant information outside of the ARWD display. This phenomena of increased focused attention to a display coinciding with decreased divided attention to an external scene is referred to as cognitive tunneling (Fischer et al., 1980). Cognitive tunneling is often implicated in aviation studies where a failure to perceive and act on an unexpected event reduces performance (Crawford and Neal, 2006).

Measurement of situation awareness and mental workload in ARWDs is problematic. Traditionally situation awareness and workload are assessed with questionnaires administered during artificial pauses (Situation Awareness Global Assessment Technique (SAGAT); Endsley, 1995b), in task probes (Situation Present Assessment Measure (SPAM); Durso and Dattel, 2004), or upon task completion NASA Task Load Index (TLX; Hart and Staveland, 1988). Within dynamic environments such assessments can be intrusive, thereby reducing ecological validity, or underrepresenting time critical signals, such as abrupt changes in workload. Workload can also be objectively assessed via dual-task secondary task decrements. In the dual task paradigm, interference on a cognitive process is anticipated between the primary task and the secondary task. This results in a decrement in performance on the secondary task, due primarily to the mental resource demands of the secondary task exceeding the mental resources that can be allocated. This secondary task performance decrement can be used as an index of the cognitive workload required of the primary task (Gopher, 1993; Wickens, 2008; Wickens et al., 2013). However, dual-task decrements have been criticized with regard to circularity; as performance varies with resource allocation, but resources are only inferred from performance (Navon, 1984).

An objective, non-invasive, motion artifact robust and portable method is needed to measure situation awareness and mental workload in ARWDs. Functional near infrared spectroscopy (fNIRS) provides an attractive method for continuous monitoring of brain dynamics in both seated and mobile participants (Ayaz et al., 2013). fNIRS is safe, highly portable, user-friendly and relatively inexpensive, with rapid application times and near-zero run-time costs (Villringer and Chance, 1997; Ayaz et al., 2012a; Ferrari and Quaresima, 2012). fNIRS uses specific wavelengths of light to provide measures of cerebral oxygenated and deoxygenated hemoglobin that are correlated with the blood-oxygen-level dependent (BOLD) contrast used in functional magnetic resonance imaging (fMRI; Cui et al., 2011; Sato et al., 2013). Importantly fNIRS measurements are objective and non-invasive to the mental task being measured. fNIRS for mobile neural measurement is also relatively robust to motion artifacts and allows wearable sensors to be physically untethered to the acquisition module (Ayaz et al., 2013; McKendrick et al., 2015). Mobile fNIRS allows for a freedom of movement not previously possible in neuroimaging, providing the opportunity to monitor mental workload and situation awareness in dynamic mobile tasks.

Hemodynamic indexes of mental workload as used by fNIRS and fMRI assume that activity related metabolic changes in specific functional brain regions are useful indexes of mental workload. Prefrontal cortex (PFC) is commonly monitored due to its functional relationship with working memory (Braver et al., 1997; Cohen et al., 1997), decision making (Ramnani and Owen, 2004; Figner et al., 2010), and executive control (Badre et al., 2005; Badre and Wagner, 2007). A growing body of research has found fNIRS hemodynamic measurements of PFC to be a useful index of mental workload in a number of complex cognitive and real world tasks (Ayaz et al., 2011, 2012b; Abibullaev and An, 2012; Naseer and Keum-Shik, 2013; Bogler et al., 2014; Derosière et al., 2014; Herff et al., 2014; Schudlo and Chau, 2014; Pinti et al., 2015; Solovey et al., 2015). Divided attention has also been associated with activity in PFC (Corbetta et al., 1991; Herath et al., 2001; Loose et al., 2003; Fagioli and Macaluso, 2009; Mizuno et al., 2012). Divided attention is a key component of dual tasking (Pashler, 1994), and superior dual-tasking has been associated with decreased activity/more efficient processing in PFC (Rypma et al., 2002; Grabner et al., 2006; McKendrick et al., 2014). Reduced demands on working memory capacity and superior dual-tasking are factors that influence greater situation awareness (Endsley, 1995a). Therefore, reduced PFC activity may be implicative of greater situation awareness during ARWD use.

The present study implemented a neuroergonomics approach (Parasuraman, 2003) to examine the cognitive differences between an ARWD (Google Glass) and a handheld display (Smartphone). We used mobile fNIRS to monitor lateral PFC and complimented it with two separate secondary tasks assessing differences in mental workload and situation awareness during navigation. Superior performance on the secondary tasks is anticipated to reflect reduced mental workload and greater situation awareness respectively. Reduced PFC activity is anticipated to index reduced mental workload and improved situation awareness in the absence of secondary task errors. Specifically, the ARWD was expected to show reduced mental workload and superior situation awareness across both behavioral and hemodynamic indices.

## Materials and Methods

### Participants

Twenty participants (12 female adults) volunteered for the study. All participants were right handed and aged 18–29 years. Each participant was randomly assigned to one of two experimental groups. The two experimental groups each contained 10 participants. If complications were experienced viewing the Google Glass display, these individuals were moved into the other experimental condition (two such complications occurred). All participants reported normal or corrected to normal vision. All participants also reported average or greater cardiovascular health, and had no history of cardiovascular abnormalities. Each participant gave informed consent via a form approved by the George Mason University Institutional Review Board prior to study participation.

### Primary Task

#### Route Following

**Figure 1:**
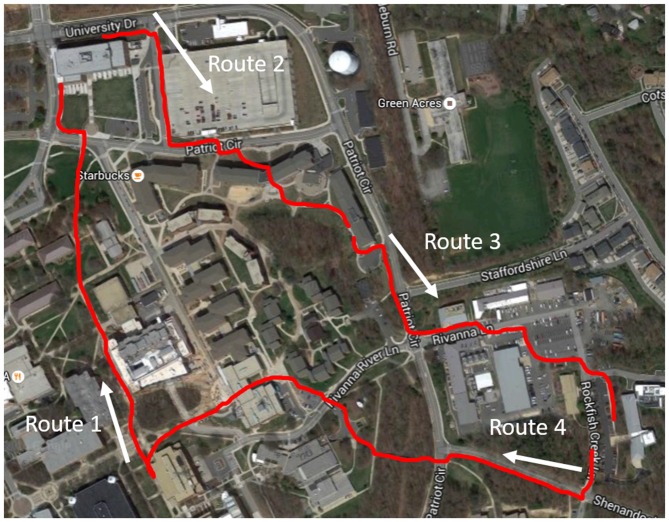
**Map depicting the four routes followed by participants.** Exact routes depicted in red, white arrows indicate walking direction. Image © 2015 DigitalGlobe.

Participants were given a visual map of a route to walk along. The visual map was generated via Google Maps and presented to the subject via an Apple Iphone 4 s (which the participant held in hand) or Google Glass (affixed to the participant’s head). In both devices Google Maps presents a birds-eye-view of the route with a digital arrow indicating the direction to be followed, as well as written turn-by-turn instructions. Google maps also provides auditory turn-by-turn instructions but these were muted in both devices. Four different routes were used (route one = 1500 ft, route two = 1400 ft, route three = 1600 ft, route four = 2000 ft; Figure 1) and each participant walked all four routes, total experiment time was between 45 and 60 min. The route following took place on a North American college campus. Portions of the routes were familiar to the participants, however the majority were unfamiliar and selected specifically because these regions are not frequented by university undergraduates. The routes also contained portions that simulated urban and rural environments. Each route was entered into either device by the experimenter. Participants in the hand held device (Smartphone; Apple Iphone 4 s) group were asked to hold the device in their right hand and lift the device near their field of view when confirmation of the correct route was needed (to avoid excessive motion artifacts in the fNIRS signal from tilting the head down). Participants in the ARWD (Google Glass) group were instructed to keep their right index finger on the Google Glass touchpad. This was done to ensure that Google Glass did not enter “sleep mode” during route following and to control for physical load in the right arm across devices. Once the route navigation began participants had no interaction with the devices other than viewing the generated route. Participants were instructed to walk at the pace they felt most comfortable with. This was done to minimize variability in the physical load of the walking task via self-adaptation. If errors were made during route following, participants were tapped on the shoulder and instructed as to the correct direction of the route. Only two such errors occurred throughout the experiment, one in each display group in the same navigation route, the error was related to a poor GPS signal.

### Secondary Tasks

#### Auditory 1-Back

While following the route, participants simultaneously completed 37 blocks of an auditory 1-back lasting 60 s each. The auditory stimuli consisted of tone triplets randomly composed from fundamental frequencies of 493.88, 554.36, 698.45 and 880 Hz presented via Bluetooth in-ear headphones. The tones were created from bandpass filtered white noise and a tone overlay. The triplets were presented randomly in one of three spatial locations; left, right and central (balanced sound distribution). Five triplets were presented for each block. Participants were asked to compare the triplet they had just heard to the triplet they had previously heard. If the two triplets were of the same frequencies presented in the same sequence, then the trial was considered a match. At the end of a block participants were prompted by the experimenter to verbally indicate how many matches they heard. The experimenter recorded the response within the program administering the auditory task and participants were immediately given feedback regarding the accuracy of their response. An fNIRS measurement block began with each 1-back block and ended just prior to the participant being prompted to respond.

#### Scenery Probe

While route following, participants were also asked 10 questions about their surroundings to assess and help maintain an accurate awareness of the environment. After a prompt from the experimenter to be “situationally aware”, participants maintained this search disposition for approximately 30 s after which the experimenter asked them to stop moving and face forward. During this time the experimenter queried whether the participant had seen a particular object in the environment. The participant was previously informed to respond verbally with a response of either “yes” or “no”. Queried objects could either have been present in the environment or not present, and there were six instances where the queried object was present and four where it was not. When the object was present in the environment the participant was stopped and queried 5 s after the object was no longer visible. Participants were given immediate feedback regarding the accuracy of their responses. The query list is presented in Table 1. An fNIRS measurement trial began when participants were prompted to be situationally aware and ended when the participant was asked to stop walking just before the scenery probe query.

**Table 1:** **List of situation awareness queries**.

Query: “Did You See”	Correct response
A drinking fountain	No
A large metallic sculpture	Yes
A children’s playground	Yes
A red “Do Not Enter” sign	No
An american flag	Yes
A black bike rack	No
A tree wrapped in multi-colored yarn	Yes
Two satellite dishes	Yes
Two figures dancing	No

### Procedures

#### fNIRS Setup

**Figure 2:**
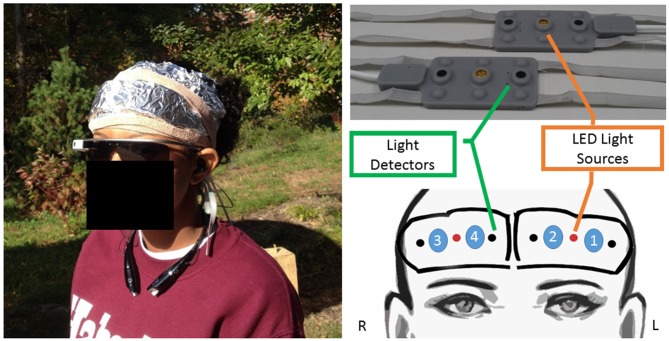
**Participant in augmented reality wearable displays (ARWD) group wearing battery operated wireless functional near infrared spectroscopy (fNIRS) sensor over the forehead, Google Glass and Bluetooth headphones (left) wireless fNIRS sensor pads (right, top) and placement sketch (right, bottom) with four optodes identified between light source and detectors**.

Participants were seated and asked to remove any makeup from their forehead with an alcohol swab and or adjust their hair prior to affixing the wireless and battery operated fNIRS neuroimaging device, Model 1100W (fNIR Devices, LLC^1^). The hardware unit was connected to headband sensor pads via cable and transmitted the data wirelessly to a remote tablet computer. Both the pocket sized control hardware (that contains the battery and antenna) and sensor pads were affixed to the subject making the participant completely mobile during recording. Two separate sensor headband pads were placed approximately 3 cm above the participant’s brow and centered approximately with respect to the eye pupil of the corresponding side, laterally symmetric from the midline of the participant’s forehead, one pad for left and the other for right hemisphere monitoring. The positioning was intended to capture hemodynamic changes in bilateral dorsolateral PFC. Draw strings attached to the sensor pads were used to prevent the pads from moving once positioned on the participant. A 9 cm wide self-adhesive bandage of length approximately the circumference of the participant’s head was folded width-wise and secured around the participant’s head across the brow just below the fNIRS sensor pads. Next a sheet of aluminum foil approximately half the circumference of the participant’s head and folded width-wise was form fitted over the bandage and fNIRS sensor pads. Care was taken to ensure that the fNIRS sensor pads were fully encapsulated by the aluminum foil sheet. This was done to ensure that while imaging in sunlight infrared light from the sun would not contaminate the fNIRS signal. Once the foil was affixed to the participant two more self-adhesive bandages of length approximately the circumference of the participant’s head were used. One bandage folded twice width-wise was wrapped around the participant’s head just below the fNIRS sensor pads, over the participant’s brow and over the aluminum foil. The second bandage was folded once width-wise and wrapped around the participant’s head just above the fNIRS sensor pads and over the foil. These bandages were used to ensure that the foil did not shift during walking, and special care was taken to minimize constrictive pressure over the fNIRS sensor as initial pilot tests showed this to be extremely uncomfortable for the participants after only a few minutes of walking. Once the sensors, foil, and bandages were positioned, the fNIRS device was turned on and the received light signal was adjusted by light source brightness and detector gain for signal quality. Also, an ambient light channel was captured to further assess signal quality. When the signal was deemed adequate, the participant was asked to put the fNIRS transmitter in their pocket. Final setup can be viewed in Figure 2.

#### Experimental Paradigm

Once the fNIRS neuroimaging setup was complete, participants were given the Bluetooth head phones and instructed to place the earbuds in their ears. Prior to this, the earbuds were cleaned with alcohol swabs. If the ear buds did not fit, a new size bud was used to optimize the setup for the participant. Once the headphones were set up, participants were introduced to the auditory 1-back and scenery probe tasks as described in sections “Auditory 1-Back” and “Scenery Probe” respectively. For the auditory task, participants were informed as to the type of stimuli they would hear, and what was considered a correct response, after which participants performed one practice block to ensure they understood the task. If participants were still unclear as to the nature of the task following the practice block, a second practice was given. No participant required more than two practice blocks in order to understand the principal of the auditory task. For the scenery probe task, participants were told they would be prompted to be “situationally aware” at which point they should be acutely aware of their surroundings. They were also informed that after being in this state for a brief period they would be questioned as to whether an object was or was not present in the environment during this time. Participants were informed that both of these tasks would take place while they were route following, but that the auditory 1-back and scenery probe task would never occur simultaneously. Once participants acknowledged they understood the nature of the two secondary tasks, the experimenters and participant relocated outdoors. All testing sessions took place between 7 and 11 am to minimize fatigue from midday heat. The participant was told they would navigate a predetermined route, the route will be displayed via a navigation device (dependent upon their group assignment) and programed into the device by the experimenter. The navigation task is described in detail in section “Route Following”. The first secondary task was prepared and the participant was instructed to begin. The secondary task orders were randomized within-subjects across the four routes, at least 15 s of navigation occurred between secondary task blocks. The start and end positions along the routes for each secondary task were preplanned so that each participant would experience the same secondary task at the same place along their navigation routes. Start and end times of the secondary tasks were synchronized with the fNIRS signal via manual entry of timing markers in the data acquisition program at the preplanned start and end positions. Upon completing a route, the participant was instructed to relax and asked whether they were still comfortable and if they wished to continue. No participant indicated they would like to stop participation due to discomfort. The navigation device was then taken by the experimenter, a new route was inputted, and the next route began.

### fNIRS Signal Processing

For each participant, raw light intensity fNIRS data (4 optodes × 2 wavelengths per optode) that were sampled at 4 Hz were low-pass filtered with a finite impulse response, linear phase filter with order 20 and cut-off frequency of 0.1 Hz to attenuate high frequency noise, respiration and cardiac cycle effects (Ayaz et al., 2011). Each participant’s data was checked for any potential saturation (when light intensity at the detector was higher than the analog-to-digital converter limit) and motion artifact contamination by means of a coefficient of variation based assessment (Ayaz et al., 2010) and for each optode, a separate channel that recorded ambient light, provided for additional verification. The light intensity changes for 730 and 850 nm wavelengths for each optode for each task block were extracted using time synchronization markers of task onset and end marked during the experiment and hemodynamic changes during each block were calculated separately using the Modified Beer-Lambert Law as described in Ayaz et al. (2012b). Ten seconds (10 s) local baselines were used in the modified Beer-Lambert law to calculate oxygenation for each task condition to look at the relative changes in oxygenated and deoxygenated hemoglobin within each task condition. The local baselines were taken at the beginning of each secondary task, during that time participants were mobile and performing the primary task. The time series for each block was further binned, the hemodynamic response at each optode across the trial was temporally divided into sub-blocks of 10 s each and each sub-block was averaged across time to provide a down-sampled hemodynamic response at each optode for each block. The final output of each optode was mean block deoxygenated hemoglobin (HbR), mean block oxygenated hemoglobin (HbO).

### fNIRS Analysis

#### Generalized and Linear Mixed Effects Models

All forthcoming statistical tests employ either linear mixed effects, or generalized linear mixed effects models implemented in R (R Core Team, 2012) via lme4 (Bates et al., 2014). Denominator degrees of freedom and *p*-values were estimated via Sattherwaite corrections implemented via lmerTest (Kuznetsova et al., 2013). These models offer several advantages as extensions of the general linear model (GLM). Such as, analysis of binomial outcomes, treatment of effects as simultaneously fixed and random, hierarchical modeling, analysis of unbalanced designs, and robustness to missing data (Pinheiro and Bates, 2000; Baayen et al., 2008; Jaeger, 2008; Verbeke and Molenberghs, 2009; Demidenko, 2013).

#### Fixed and Random Effects Selection

Bayesian information criterions was used to select the fixed and random effects in the final models for each dependent variable. Competing models were constructed by adding potentially meaningful random and fixed effects to a null model. The null model was specified in each case as having no fixed effects and a random effect of participant intercept. All competing models were estimated with maximum likelihood to allow for testing of fixed effects. The competing models were tested simultaneously with BIC and the strength of evidence criterion described by Kass and Raftery (1995) was employed. In the procedure deviations of greater than two BIC are viewed as a meaningful difference. The final model was selected based on having the lowest BIC, with no other models of interest having a BIC deviance of less than two. This procedure serves to both minimize over fitting of the models random effects, and to act as an omnibus test of variance for fixed effects and interactions between fixed effects, as passing this procedure ensures that these variables accounted for a meaningful amount of variance in the data.

#### Multiple Comparisons Corrections

In all forthcoming analyses of fNIRS data multiple comparisons were corrected for across hypotheses and optodes but within secondary tasks and chromophores by adjusting *p*-value criterion with false discovery rate (FDR) corrections. Controlling for FDR can increase statistical power relative to correcting for multiple comparisons via controlling for the familywise error rate (FWER). The Benjamini-Hockberg FDR procedure, employed here for controlling the FDR is adaptive in that the threshold for rejecting the null hypothesis is dependent on the size of the initial *p*-value and the number of hypotheses tested (Benjamini and Hochberg, 1995; Lindquist, 2008). Adjustments were made with alpha set to 0.05 in the Benjamini-Hockberg equation.

## Results

### Auditory 1-Back

#### Behavioral

**Figure 3:**
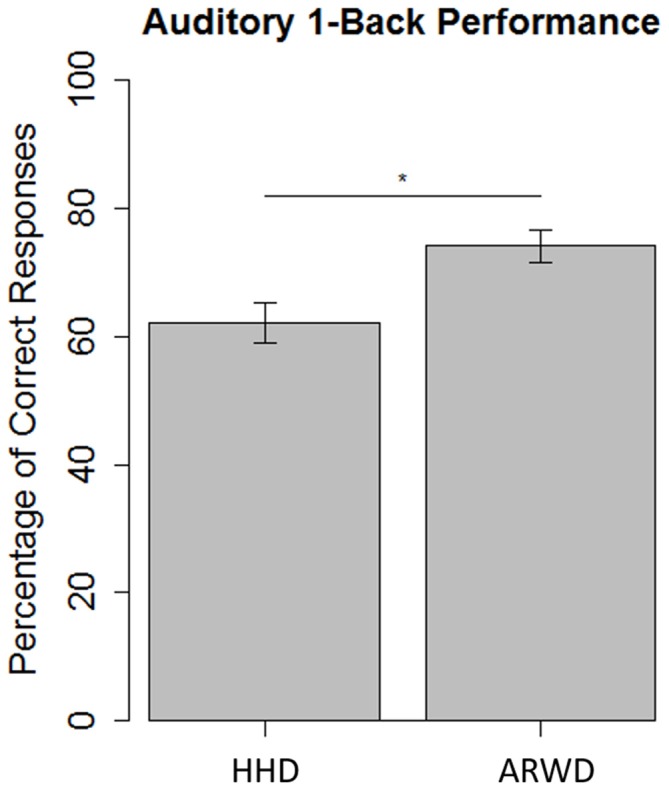
**Percentage of correct responses during an auditory 1-back while navigating with an ARWD or a HHD.** *p < 0.05.

The results of the auditory 1-back were submitted to a generalized linear mixed effects regression. The link function was specified as binomial and parameter estimates were calculated using maximum likelihood. The tested fixed effects included condition (ARWD vs. HHD with smartphone coded as the reference factor), trial and the interaction between the two. The trial component was included to determine if there were any accommodation effects within and between the two devices. Participant intercepts were specified as the random effect. Parameter estimates are reported here as log odds ratios (as they are linear and non-conditional within this analysis). Participants in the HHD group were more likely to correctly than incorrectly report the number of matches heard (*b* = 0.528, *SE* = 0.178, *p* < 0.005). Participants in the ARWD group were more likely than the HHD group to correctly report the number of matches heard (*b* = 0.551, *SE* = 0.257, *p* < 0.05; Figure 3). The effect of trial was non-significant (*b* = 0.019, *SE* = 0.013, *p* = 0.137), and this did not differ between the two device groups (*b* = 0.013, *SE* = 0.019, *p* = 0.492). These results suggest that participants in the ARWD condition experienced lower levels of cognitive load relative to participants in the HHD condition when route following. Furthermore, there is no evidence that this level of load changed throughout the experiment both within and between conditions.

#### fNIRS

Relative measures of HbO and HbR acquired during the auditory 1-back were submitted to a linear mixed effects regression. Parameter estimates in the model selected from the procedure described in section “Fixed and Random Effects Selection” were calculated with restricted maximum likelihood. Fixed effects were condition (ARWD vs. HHD) and an interaction with performance (correct vs. incorrect). Participant intercepts and block slope were specified as random effects for HbO, and participant block slope was specified for HbR. The results of models for optodes over left lateral, left medial, right medial, and right lateral PFC are reported in Tables 2–5.

**Table 2:** **Auditory 1-back secondary-task hemodynamics as a function of device and accuracy**.

	Left lateral prefrontal cortex			
	HbO	HbR
	*B*	*t*-value	*B*	*t*-value
**Fixed**
Correct	−0.298**	−2.88	−0.064	−1.76
Incorrect	−0.118	−1.00	−0.008	−0.17
Incorrect-Correct	0.180**	2.60	0.056	1.39
ARWD	−0.252	−1.82	−0.023	−0.48
HHD	−0.164	−1.03	−0.050	−0.87
HHD-ARWD	0.088	0.42	−0.027	−0.36
ARWD: Correct	−0.486**	−3.54	−0.001	−0.02
ARWD: Incorrect	−0.018	−0.12	−0.045	−0.74
ARWD: Incorrect-Correct	0.469***	5.38	−0.044	−0.87
HHD: Correct	−0.110	−0.71	−0.128	−2.29
HHD: Incorrect	−0.218	−1.21	0.029	0.39
HHD: Incorrect-Correct	−0.108	−1.01	0.157*	2.49
HHD: Correct-ARWD:	0.376	1.82	−0.127	−1.73
Correct
HHD: Incorrect-ARWD:	−0.201	−0.85	0.074	0.77
Incorrect

	*Var*	*Std. Dev*	*Var*	*Std. Dev*

**Random**
Intercept	0.150	0.387
Block slope	0.002	0.048	0.008	0.090
Residual	1.102	1.050	0.327	0.610

Notes. *p < 0.05; **p < 0.01; ***p < 0.001.

**Table 3:** **Auditory 1-back secondary-task hemodynamics as a function of device and accuracy**.

	Left medial prefrontal cortex			
	HbO		HbR	
	*B*	*t*-value	*B*	*t*-value
**Fixed**
Correct	−0.087	−0.77	−0.201***	−3.82
Incorrect	0.223	1.78	−0.029	−0.42
Incorrect-Correct	0.310***	4.17	0.172**	2.73
ARWD	0.063	0.46	−0.092	−1.46
HHD	0.074	0.41	−0.137	−1.68
HHD-ARWD	0.011	0.05	−0.045	−0.44
ARWD: Correct	−0.240	−1.74	−0.113	−1.77
ARWD: Incorrect	0.366*	2.42	−0.071	−0.87
ARWD: Incorrect-Correct	0.607***	6.83	0.042	0.55
HHD: Correct	0.067	0.38	−0.288***	−3.45
HHD: Incorrect	0.080	0.40	0.014	0.13
HHD: Incorrect-Correct	0.013	0.11	0.303**	3.01
HHD: Correct-ARWD:	0.308	1.37	−0.176	−1.67
Correct
HHD: Incorrect-ARWD:	−0.286	−1.14	0.086	0.64
Incorrect

	*Var*	*Std. Dev*	*Var*	*Std. Dev*

**Random**
Intercept	0.126	0.354
Block slope	0.006	0.080	0.002	0.043
Residual	0.876	0.936	0.654	0.809

Notes. *p < 0.05; **p < 0.01; ***p < 0.001.

**Table 4:** **Auditory 1-back secondary-task hemodynamics as a function of device and accuracy**.

	Right medial prefrontal cortex			
	HbO		HbR	
	*B*	*t*-value	*B*	*t*-value
**Fixed**
Correct	−0.111	−0.80	−0.061	−1.24
Incorrect	0.181	1.19	0.005	0.08
Incorrect-Correct	0.292***	3.52	0.066	1.14
ARWD	0.010	0.50	0.013	0.19
HHD	−0.029	−0.15	−0.069	−1.00
HHD-ARWD	−0.129	−0.46	−0.082	−0.84
ARWD: Correct	−0.195	−0.99	0.016	0.23
ARWD: Incorrect	0.395	1.83	0.010	0.11
ARWD: Incorrect-Correct	0.590***	4.92	−0.006	−0.07
HHD: Correct	−0.026	−0.13	−0.137	−2.00
HHD: Incorrect	−0.032	−0.15	−0.001	−0.01
HHD: Incorrect-Correct	−0.006	−0.05	0.137	1.72
HHD: Correct-ARWD:	0.169	0.61	−0.153	−1.57
Correct
HHD: Incorrect-ARWD:	−0.427	−1.40	−0.011	−0.08
Incorrect

	*Var*	*Std. Dev*	*Var*	*Std. Dev*

**Random**
Intercept	0.192	0.438		
Block Slope	0.001	0.027	0.003	0.053
Residual	0.975	0.987	0.473	0.688

Notes.***p < 0.001.

**Table 5:** **Auditory 1-back secondary-task hemodynamics as a function of device and accuracy**.

	Right lateral prefrontal cortex			
	HbO		HbR	
	*B*	*t*-value	*B*	*t*-value
**Fixed**
Correct	−0.287***	−5.23	−0.065	−1.98
Incorrect	−0.122	−1.73	−0.064	−1.45
Incorrect-Correct	0.165**	2.76	0.001	0.03
ARWD	−0.156	−1.95	0.058	1.24
HHD	−0.252**	−3.28	−0.186***	−4.18
HHD-ARWD	−0.097	−0.87	−0.245***	−3.77
ARWD: Correct	−0.366***	−4.68	0.066	1.44
ARWD: Incorrect	0.055	0.54	0.050	0.78
ARWD: Incorrect-Correct	0.421***	4.89	−0.016	−0.27
HHD: Correct	−0.207*	−2.69	−0.196***	−4.24
HHD: Incorrect	−0.298**	−3.08	−0.177**	−2.99
HHD: Incorrect-Correct	−0.091	−1.10	0.019	0.32
HHD: Correct-ARWD:	0.159	1.45	−0.262***	−4.01
Correct
HHD: Incorrect-ARWD:	−0.353*	−2.51	−0.227**	−2.60
Incorrect

	*Var*	*Std. Dev*	*Var*	*Std. Dev*

**Random**
Intercept	0.001	0.001		
Block Slope	0.003	0.054	0.001	0.025
Residual	0.707	0.841	0.352	0.594

Notes. *p < 0.05; **p < 0.01; ***p < 0.001.

##### Left Lateral PFC (LLPFC)

Correct blocks while using an ARWD were associated with a decrease in the hemodynamic response as evidenced by a reduction in oxygenated hemoglobin relative to the null hypothesis. Furthermore, correct blocks while using a HHD were associated with an increase in the hemodynamic response as evidenced by a decrease in deoxygenated hemoglobin relative to incorrect blocks.

##### Left Medial PFC (LMPFC)

Correct blocks while using a HHD were associated with an increase in the hemodynamic response as evidenced by a reduction in deoxygenated hemoglobin. Furthermore, there is evidence to suggest that incorrect blocks while using an ARWD were associated with an increase in the hemodynamic response. Specifically, relative to the null hypothesis and correct blocks, incorrect blocks were related to an increase in oxygenated hemoglobin.

##### Right Medial PFC (RMPFC)

Incorrect bloks while using an ARWD were associated with an increase in the hemodynamic response as evidenced by increased oxygenated hemoglobin relative to correct blocks.

##### Right Lateral PFC (RLPFC)

Correct bloacks while using an ARWD were associated with a decrease in the hemodynamic response as evidenced by reduced oxygenated hemoglobin relative to the null hypothesis and incorrect blocks. Furthermore, HHD use during correct and incorrect blocks reduced total hemoglobin as evidenced by the reductions in oxy and deoxygenated hemoglobin. Of particular note for workload comparison between the display conditions is that during correct blocks HHD deoxygenated hemoglobin was less than ARWD deoxygenated hemoglobin. Finally, during incorrect blocks HHD use was associated with decreases in oxygenated and deoxygenated hemoglobin relative to ARWD use.

**Figure 4:**
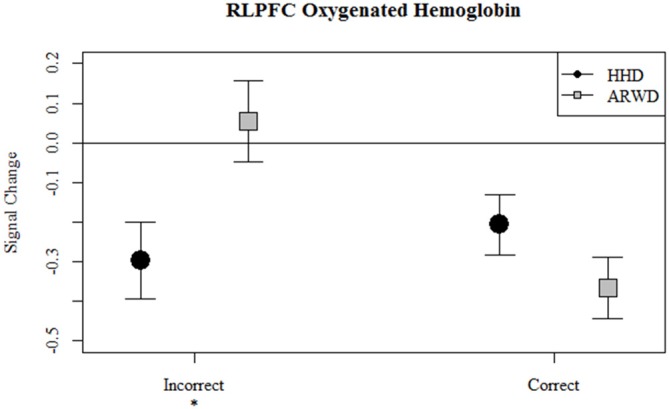
**Relative concentrations of oxygenated hemoglobin in RLPFC for correct and incorrect blocks of an auditory 1-back while navigating with an ARWD (Google Glass) and HHD (Smartphone).** *p < 0.05.

**Figure 5:**
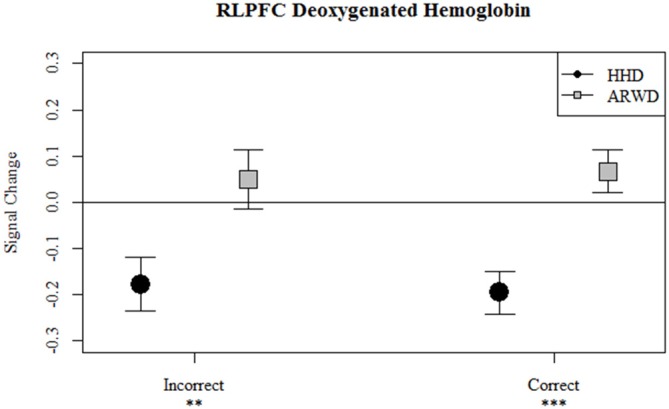
**Relative concentrations of deoxygenated hemoglobin in RLPFC for correct and incorrect blocks of an auditory 1-back while navigating with an ARWD (Google Glass) and HHD (Smartphone).** **p < 0.01; ***p < 0.001.

Overall, correct auditory memory performance while using ARWD was associated with a reduction in the hemodynamic response in bilateral PFC. Interestingly, incorrect responses were associated with an increase in the hemodynamic response at the more medial measurement sites. Effects of HHD use were mainly observed in left medial PFC, where correct auditory memory performance while using a HHD was associated with an increase in the hemodynamic response. Workload differences as inferred from errors on secondary tasks are most apparent in RLPFC. Where auditory errors during HHD use were associated with reductions in oxygenated (Figure 4) and deoxygenated (Figure 5) hemoglobin relative to ARWD use.

### Situation Awareness

#### Behavioral

**Figure 6:**
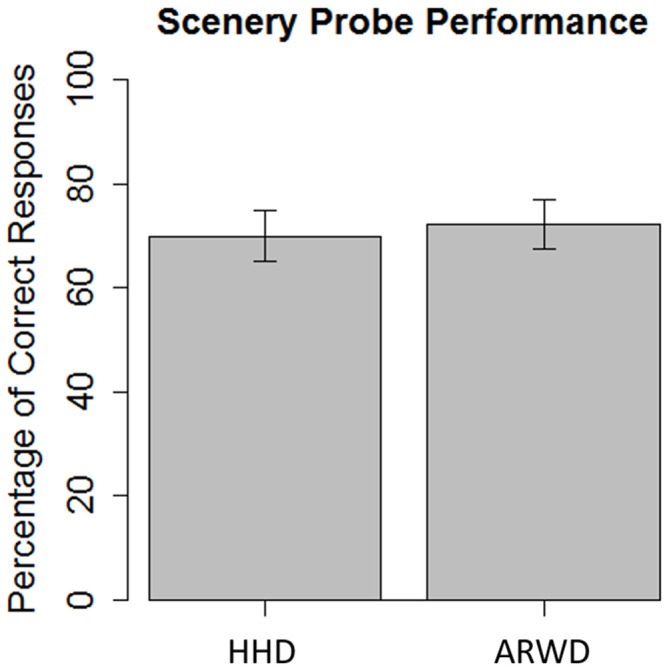
**Percentage of correct responses during a scenery probe task while navigating with ARWD or a HHD**.

The results of the scenery probe task were submitted to a generalized linear mixed effects regression. The link function was specified as binomial and parameter estimates were calculated using maximum likelihood. The tested fixed effects were condition (ARWD vs. HHD with HHD coded as the reference factor), trial and the interaction between the two. The trial component was included to determine if there were any accommodation effects within and between the two devices. Participant intercepts and uncorrelated trial slopes were specified as the random effects. Parameter estimates are reported here as log odds ratios. Participants in the HHD group were more likely to correctly respond to the scenery probe (*b* = 0.914, *SE* = 0.235, *p* < 0.001) than not. Participants in the ARWD group showed no significant difference relative to the HHD group in correctly responding to the scenery probe (*b* = −0.155, *SE* = 0.335, *p* = 0.644; Figure 6). The effect of trial was non-significant (*b* = −0.098, *SE* = 0.091, *p* = 0.282), and this did not differ between the two device groups (*b* = −0.148, *SE* = 0.132, *p* = 0.260). These results suggest that participants in the both conditions were able to effectively perform the task. However, there is no measureable difference in situation awareness for environmental objects between the two conditions. Furthermore, there is no evidence that situation awareness changed throughout the experiment both within and between conditions.

#### fNIRS

Relative measures of HbO and HbR acquired during the scenery probe task were submitted to a linear mixed effects regression. Parameter estimates in the model selected from the procedure described in section “Fixed and Random Effects Selection” were calculated with restricted maximum likelihood. Fixed effects were condition (ARWD vs. HHD) and an interaction with performance (correct vs. incorrect). Participant random trial slopes were specified as random effects. The results of models for optodes over left lateral, left medial, right medial, and right lateral PFC are reported in Tables 6–9.

**Table 6:** **Scenery probe secondary-task hemodynamics as a function of device and accuracy**.

	Left lateral prefrontal cortex			
	HbO		HbR	
	*B*	*t*-value	*B*	*t*-value
**Fixed**
Correct	−0.165**	−2.82	−0.051	−1.67
Incorrect	0.008	0.10	−0.096	−2.36
Incorrect-Correct	0.173	2.18	−0.045	−1.19
ARWD	0.015	0.19	0.017	0.44
HHD	−0.172	−1.94	−0.164***	−3.53
HHD-ARWD	−0.187	−1.59	−0.181**	−2.97
ARWD: Correct	−0.238**	−3.18	−0.004	−0.11
ARWD: Incorrect	0.268*	2.45	0.039	0.72
ARWD: Incorrect-Correct	0.506***	4.80	0.043	0.85
HHD: Correct	−0.092	−1.02	−0.098	−2.08
HHD: Incorrect	−0.252	−2.09	−0.230***	−3.78
HHD: Incorrect-Correct	−0.160	−1.35	−0.133	−2.34
HHD: Correct-ARWD:	0.146	1.25	−0.094	−1.54
Correct
HHD: Incorrect-ARWD:	−0.520**	−3.19	−0.269***	−3.31
Incorrect

	*Var*	*Std. Dev*	*Var*	*Std. Dev*

**Random**
Trial slope	0.009	0.097	0.005	0.072
Residual	0.568	0.753	0.128	0.358

Notes. *p < 0.05; **p < 0.01; ***p < 0.001.

**Table 7:** **Scenery probe secondary-task hemodynamics as a function of device and accuracy**.

	Left medial prefrontal cortex			
	HbO	
	*B*	*t*-value	*B*	*t*-value
**Fixed**
Correct	−0.146	−2.02	−0.080	−1.34
Incorrect	−0.172	−1.58	−0.168	−1.94
Incorrect-Correct	−0.026	−0.24	−0.087	−1.11
ARWD	−0.155	−1.68	−0.038	−0.49
HHD	−0.163	−1.38	−0.210	−2.09
HHD-ARWD	−0.008	−0.05	−0.173	−1.37
ARWD: Correct	−0.218*	−2.60	−0.107	−1.52
ARWD: Incorrect	−0.092	−0.66	0.032	0.29
ARWD: Incorrect-Correct	0.125	0.91	0.139	1.38
HHD: Correct	−0.074	−0.63	−0.054	−0.55
HHD: Incorrect	−0.251	−1.51	−0.367**	−2.73
HHD: Incorrect-Correct	−0.176	−1.07	−0.313*	−2.58
HHD: Correct-ARWD:	0.021	0.24	0.053	0.44
Correct
HHD: Incorrect-ARWD:	−0.158	−0.73	−0.399	−2.31
Incorrect

	*Var*	*Std. Dev*	*Var*	*Std. Dev*

**Random**
Trial slope	0.006	0.079	0.011	0.105
Residual	0.637	0.798	0.336	0.579

Notes. *p < 0.05; **p < 0.01.

**Table 8:** **Scenery probe secondary-task hemodynamics as a function of device and accuracy**.

	Right medial prefrontal cortex			
	HbO		HbR	
	*B*	*t*-value	*B*	*t*-value
**Fixed**
Correct	−0.134	−1.98	−0.044	−0.80
Incorrect	−0.118	−1.20	−0.002	−0.02
Incorrect-Correct	0.016	0.16	0.042	0.50
ARWD	−0.080	−0.79	0.077	0.91
HHD	−0.172	−1.77	−0.122	−1.54
HHD-ARWD	−0.091	−0.65	−0.199	−1.72
ARWD: Correct	−0.141	−1.50	−0.041	−0.54
ARWD: Incorrect	−0.019	−0.13	0.194	1.53
ARWD: Incorrect-Correct	0.122	0.86	0.236	1.87
HHD: Correct	−0.126	−1.30	−0.047	−0.59
HHD: Incorrect	−0.217	−1.65	−0.198	−1.76
HHD: Incorrect-Correct	−0.091	−0.72	−0.151	−1.35
HHD: Correct-ARWD:	0.015	0.11	−0.006	−0.05
Correct
HHD: Incorrect-ARWD:	−0.197	−1.00	−0.392	−2.31
Incorrect

	*Var*	*Std. Dev*	*Var*	*Std. Dev*

**Random**
Trial slope	0.012	0.108	0.004	0.067
Residual	0.448	0.669	0.360	0.600

**Table 9:** **Scenery probe secondary-task hemodynamics as a function of device and accuracy**.

	Right lateral prefrontal cortex			
	HbO		HbR	
	*B*	*t*-value	*B*	*t*-value
**Fixed**
Correct	−0.045	−1.04	−0.060	−1.56
Incorrect	−0.114	−1.98	−0.100	−1.92
Incorrect-Correct	−0.069	−1.28	−0.039	−0.79
ARWD	0.043	0.75	−0.054	−1.03
HHD	−0.2019**	−3.17	−0.106	−1.86
HHD-ARWD	−0.2453**	−2.85	−0.053	−0.68
ARWD: Correct	−0.055	−0.95	0.017	0.32
ARWD: Incorrect	0.142	1.83	−0.124	−1.75
ARWD: Incorrect-Correct	0.1969**	2.65	−0.141	−2.06
HHD: Correct	−0.034	−0.54	−0.137	−2.41
HHD: Incorrect	−0.3696***	−4.37	−0.076	−0.99
HHD: Incorrect-Correct	−0.3354***	−4.28	0.062	0.86
HHD: Correct-ARWD:	0.021	0.24	−0.154	−1.99
Correct
HHD: Incorrect-ARWD:	−0.5114***	−4.45	0.048	0.46
Incorrect

	*Var*	*Std. Dev*	*Var*	*Std. Dev*

**Random**
Trial slope	0.008	0.091	0.005	0.073
Residual	0.216	0.464	0.182	0.427

Notes. **p < 0.01; ***p < 0.001.

##### Left Lateral PFC

Correct trials while using an ARWD, were associated with a decrease in oxygenated hemoglobin. Incorrect ARWD trials were associated with an increase in oxygenated hemoglobin, and the difference in relative oxygenated hemoglobin between the two outcomes was significant. Incorrect trials while using a HHD were related to reduced deoxygenated hemoglobin. Finally, incorrect trials while using a HHD reduced oxygenated and deoxygenated hemoglobin relative to incorrect trials while using an ARWD. This is either representative of only a reduction in total hemoglobin or a reduction in total hemoglobin and a reduction in brain activity in this region as the decline in oxygenated hemoglobin is greater than that of deoxygenated hemoglobin.

##### Left Medial PFC

Correct trials while using an ARWD were associated with a decrease in the hemodynamic responses as evidenced by the reduction in oxygenated hemoglobin. Furthermore, incorrect trials while using a HHD were associated with an increase in the hemodynamic response as evidenced by reduced deoxygenated hemoglobin.

##### Right Medial PFC

No significant differences in hemodynamics were observed in regards to accuracy, or device use.

##### Right Lateral PFC

Incorrect trials while using a HHD were associated with a decrease in the hemodynamic response as evidenced by reduced oxygenated hemoglobin relative to the null hypothesis and correct trials. Furthermore, incorrect trials while using an ARWD were associated with an increase in the hemodynamic response as evidenced by an increase in oxygenated hemoglobin relative to correct trials, and incorrect trials while using a HHD.

**Figure 7:**
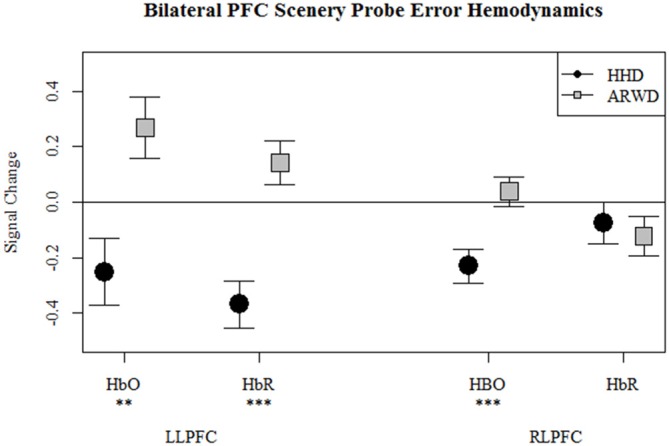
**Relative oxygenated and deoxygenated hemoglobin at bilateral optodes for incorrect trials of the scenery probe task while navigating with an ARWD (Google Glass) and HHD (Smartphone).** **p < 0.01, ***p < 0.001.

Overall, high situation awareness while using glass was associated with a reduced hemodynamic response in left PFC. Low situation awareness while using glass was related to an increase in the hemodynamic response in bilateral PFC. Conversely, low situation awareness while using a smartphone was associated with a reduced hemodynamic response in bilateral PFC (Figure 7).

## Discussion

ARWDs are increasing in use and it is important that we understand how such devices affect mental workload and situation awareness. NASA plans to use ARWDs to improve training and performance in highly demanding situations (Schierholz et al., 2015). Objectively measuring mental workload and situation awareness in ARWDs can be difficult due to the immersive and mobile nature of the technology. To circumvent issues of mobility and immersion we used wireless fNIRS to examine hemodynamic differences in mental workload and situation awareness between an ARWD (i.e., Google Glass) and a hand-held display (i.e., a smartphone) during real-world navigation and dual-tasking.

Behavioral differences between the ARWD and HHD while navigating and performing an auditory working memory task suggest differences in experienced workload. While dual-tasking, both tasks were preformed successfully across displays types. However, individuals using an ARWD showed superior working memory recall relative to HHD users. The dual-task method of assessing mental workload (Ogden et al., 1979; O’Donnell and Eggemeier, 1986) dictates that higher performance observed in secondary tasks represents reduced workload during the primary task. The increased working memory performance observed while using an ARWD suggests that relative to hand-held displays ARWDs induce less mental workload while being used for navigation.

Mental workload and the hemodynamic response representative of brain activity are positively related, especially in working memory tasks (Braver et al., 1997; Cohen et al., 1997; Culham et al., 2001; Ayaz et al., 2012b); from our behavioral results, we expected a lower hemodynamic response for ARWD users relative to HHD users. In accordance with our behavioral results ARWD blocks were associated with a reduction of oxygenated hemoglobin representative of a reduction of brain activity in bilateral PFC. Furthermore, HHD trials were associated with a reduction of deoxygenated hemoglobin representative of an increase in brain activity in left medial and right lateral PFC. A direct comparison of the two conditions hemodynamics in RLPFC revealed reduced deoxygenated hemoglobin during HHD use relative to ARWD use during correct auditory working memory performance. This provides further evidence that even when the interference between the auditory working memory task and the navigation task was not *overloading*, neural activity was higher while using an HHD.

With regard to the scenery probe task, we observed no performance differences between ARWDs and hand-held displays, but hemodynamic differences were observed. Both display groups performed the scenery probe and navigation tasks successfully. However, unlike when working memory and navigation co-occurred, dual-task assessment could not differentiate between the two displays in terms of mental workload during the scenery probe task. This was not the case for hemodynamic measurements made with wireless fNIRS. The difference between the display conditions is strongest in left lateral PFC. In this region there was a reduction in oxygenated hemoglobin during ARWD use on correct trials. A decrement was not present in left lateral PFC during hand-held display use. While not as large, a similar trend can be seen between ARWD and hand-held displays in right lateral PFC as well. While inconclusive, considering the non-significant differences in oxygenated hemoglobin on correct trials between ARWD and hand-held displays, the trend is for reduced brain activity during ARWD use. Taking the scenery probe task as a proxy for level 1 and 2 situation awareness, less mental resources were required during landmark perception and comprehension while navigating with an ARWD relative to a hand-held display.

Scenery probe and working memory errors were associated with changes in ARWD hemodynamics. Lower situation awareness during ARWD use was associated with increased oxygenated hemoglobin in bilateral PFC. Similarly, incorrect working memory trials and ARWD use were associated with increased oxygenated hemoglobin across PFC. Effectively, poor secondary task performance was associated with an increase in PFC activity while navigating with an ARWD. This increase in activity coincides with the increase in workload expected due to dual-task interference. Stimulus driven attention capture is related to increased activity in PFC (Fockert et al., 2004; Serences et al., 2005; Asplund et al., 2010). Furthermore, head-up display symbology is known to negatively affect performance from unnecessary attention capture (Thomas and Wickens, 2001, 2004). The presence of cognitive tunneling during ARWD use can parsimoniously explain the presence of an error, the increase in brain activity and the increase in mental workload observed across both secondary tasks. Also considering that the display symbology was unchanged between the ARWD and HHD conditions, and that the symbology was originally designed for the HHD; the presence of cognitive tunneling was expected.

The association of secondary task errors on HHD hemodynamics was the opposite of that observed during ARWD use. Across both secondary tasks, errors were associated with decreases in brain activity. Working memory errors were associated with an increase in left PFC deoxygenated hemoglobin. Lower situation awareness was associated with a decrease in bilateral PFC oxygenated hemoglobin and RLPFC deoxygenated hemoglobin. It is probable that HHD errors were related to task shedding, the abandonment of one of the two tasks being performed; a common strategy during dual-tasks that overload mental resources (Schneider and Detweiler, 1988; Raby and Wickens, 1994; Hancock and Szalma, 2003; Grier et al., 2008; Schulte and Donath, 2011). Task shedding should produce a reduction in brain activity due to reducing mental workload. Therefore, we would expect a reduced hemodynamic response during correct secondary task trials if the primary task was shed. This effect was not observed. Instead, brain activity decreased during incorrect trials. Continuing with the logic that reduced activity is related to reduced mental workload, reduced activity during incorrect secondary trials suggests that the secondary-tasks may have been shed. This explanation is consistent with the emphasis we placed on the navigation task as well as our observed behavioral and hemodynamic effects.

## Limitations

Due to the nature of the wireless fNIRS, and the miniaturized design of our imaging unit we are limited to four optodes imaging the PFC. Therefore, other cortical regions may have shown significant hemodynamic differences between the two devices that we could not measure. Furthermore, given the current design, we could not account for all factors that might influence difference in mental workload between the two devices. We could only measure differences in mental workload that manifest as dual-task interference from increased working memory load, or increased perceptual load.

## Conclusion

Taking a neuroergonomic approach combining dual-task interference and wireless fNIRS, we were able to examine differences in mental workload, and situation awareness between a hand-held display (smartphone) and an augmented reality wearable display (Google Glass) while navigating an outdoor environment. ARWDs show few downsides with regards to dual tasking while route following. Relative to a HHD, mental workload while navigating with an ARWD was reduced, both during a working memory and situation awareness secondary task; performance was also enhanced during the working memory dual-task. Hemodynamic effects induced during errors also suggest ways in which ARWDs can be improved, specifically by reducing unwanted attention capture and cognitive tunneling. Future work should identify other hemodynamic biomarkers induced by cognitive tunneling. From an applied perspective development of tunneling biomarkers could greatly advance display design for navigation, training and other tasks ARWDs are expected to enhance.

## Dedication

In Memory of Professor Raja Parasuraman, this article is dedicated to Professor Parasuraman for his guidance on past and present neuroergonomic studies as well as the inspiration he provides for studies to come.

## Author Contributions

RMcK designed the study, collected the data, analyzed the data and wrote the manuscript. RP designed the study. RM designed the study and collected data. AF designed the study and collected data. WB designed the study, analyzed data and edited the manuscript. MP analyzed data and edited the manuscript. HA designed the study, analyzed data and wrote the manuscript.

## Funding

This research was supported by Air Force Office of Scientific Research (AFOSR) Grant No. FA9550-10-1-0385, and the Center of Excellence in Neuroergonomics, Technology, and Cognition (CENTEC).

## Conflict of Interest Statement

fNIR Devices, LLC manufactures the optical brain imaging instrument and licensed IP and know-how from Drexel University. HA was involved in the technology development and thus offered a minor share in the new startup firm fNIR Devices, LLC. The authors declare that the research was conducted in the absence of any commercial or financial relationships that could be construed as a potential conflict of interest. The Review Editor SC and handling Editor declared their shared affiliation, and the handling Editor states that the process nevertheless met the standards of a fair and objective review.

## Footnotes

^1^www.fnirdevices.com.
